# Role of IKKε in the Metabolic Diseases: Physiology, Pathophysiology, and Pharmacology

**DOI:** 10.3389/fphar.2022.888588

**Published:** 2022-05-19

**Authors:** Qing-Ao Xiao, Qian He, Lun Li, Yinhong Song, Yue-ran Chen, Jun Zeng, Xuan Xia

**Affiliations:** ^1^ Department of Endocrinology, The People’s Hospital of China Three Gorges University/the First People’s Hospital of Yichang, Yichang, China; ^2^ Third-grade Pharmacological Laboratory on Traditional Chinese MedicineState Administration of Traditional Chinese Medicine, China Three Gorges University, Yichang, China; ^3^ National Clinical Research Center for Metabolic Diseases, Hunan Provincial Key Laboratory of Metabolic Bone Diseases, Department of Metabolism and Endocrinology, The Second Xiangya Hospital of Central South University, Changsha, China; ^4^ The Institute of Infection and Inflammation, China Three Gorges University, Yichang, China; ^5^ Department of Microbiology and Immunology, Medical College, China Three Gorges University, Yichang, China; ^6^ Department of Physiology and Pathophysiology, Medical College, China Three Gorges University, Yichang, China

**Keywords:** inhibitor of nuclear factor kappa-B kinase ε, nonalcoholic fatty liver disease, diabetes, obesity, metabolic diseases, amlexanox

## Abstract

IKKε (inhibitor of nuclear factor kappa-B kinase ε) is a member of the noncanonical NF-κB pathway. It participates in the inflammatory response and innate immunity against bacteria. In recent decades, IKKε has been closely associated with metabolic regulation. Inhibition of the IKKε pathway can improve fat deposition in the liver, reduce subcutaneous fat inflammation, and improve liver gluconeogenesis in obesity. IKKε is expected to be a new therapeutic target for metabolic diseases such as nonalcoholic fatty liver disease, diabetes, and obesity. Herein, we summarize the structural characterization, physiological function, and pathological role of IKKε in metabolic diseases and small molecule inhibitors of IKKε.

## 1 Introduction

IKKε (inhibitor of nuclear factor kappa-B kinase ε), also named IKK-inducible kinase, IKKe, IKBKE, and IKK-i, belongs to the noncanonical IKK family which consists of IKKε and TANK binding kinase 1 (TBK1). In 1999, Shimada et al. (1999) discovered IKKε from the mouse macrophage (RAW 264.7 cell), which was induced by lipopolysaccharides (LPS) and then phosphorylated serine residues (Ser32 and Ser36) of IκB-α, resulting in NF-κB activation. Recent studies have shown that energy adjustment disorders are closely related to widespread and low inflammation involving the classical NF-κB pathway (Yuan et al., 2001; Cai et al., 2005).

The role of IKKε in energy regulation was not revealed until 2009, and the expression of IKKε in the liver, adipocytes, and macrophages in the adipose tissue was induced by a high-fat diet in the mice. IKKε^−/−^ mice were resistant to HFD-induced obesity and chronic inflammation in the adipose tissue and improved insulin sensitivity (Chiang et al., 2009). Recently, IKKε has been associated with obesity, diabetes, and nonalcoholic fatty liver disease (NAFLD), and IKKε inhibition suppresses inflammation and increases energy expenditure and thermogenesis. This review focuses on the biochemical structure, physiological function, regulation, and pathological role of IKKε and its inhibitors function.

## 2 Biochemical Structure of IKKε

### 2.1 Structure and Function of IKKε

**FIGURE 1:**
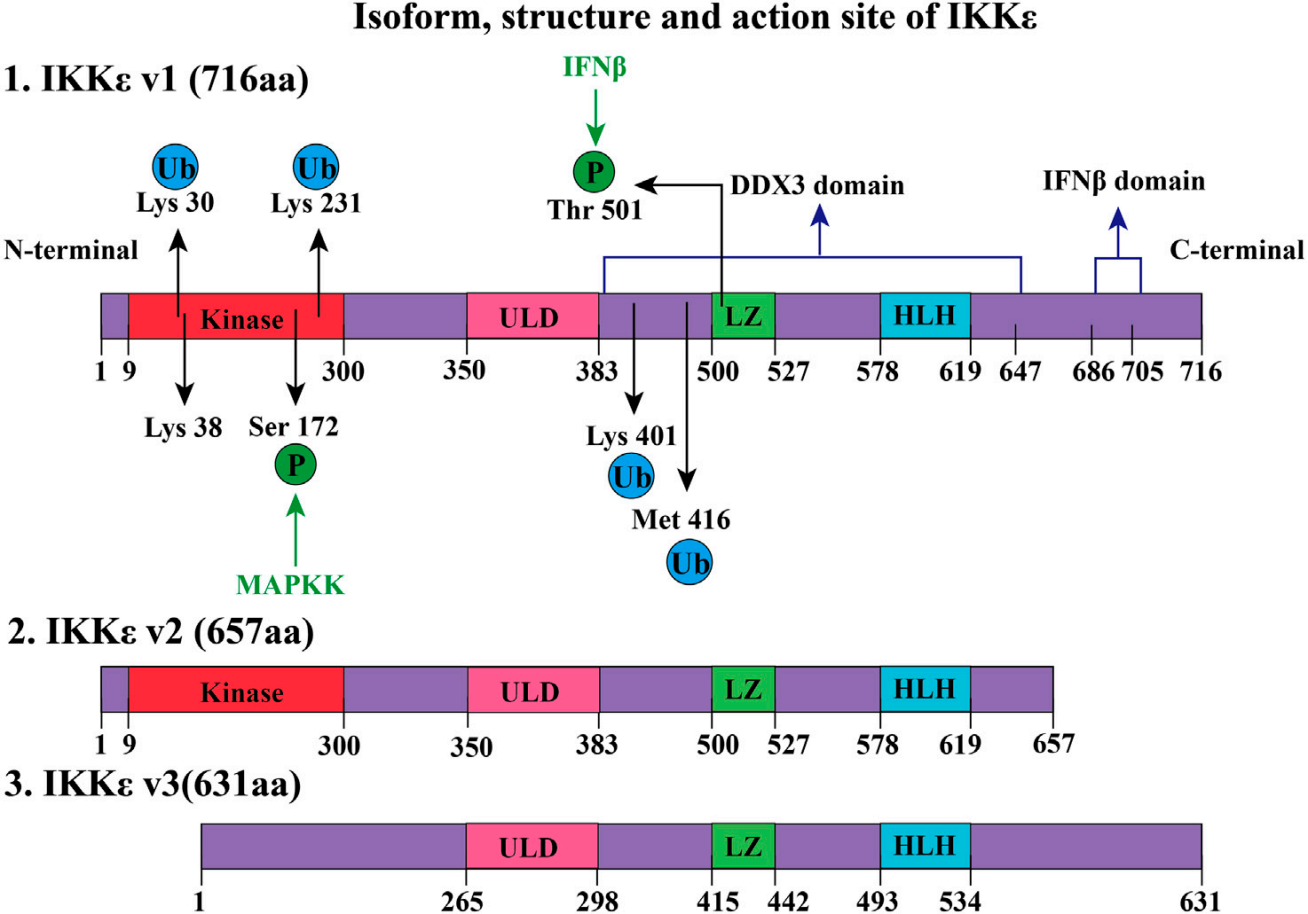
Isoform, structure, and active site of IKKε. IKKε exists in three forms, namely, IKKε v1, IKKε v2, and IKKε v3. IKKε v1 is a full-length protein and has 716 amino acids (aa). IKKε v1 contains several regions: 1) a kinase domain in its N-terminal region, is composed of amino acids 9–300 and is a key site of ATP binding. 2) Amino acids at positions 350–383 constitute the ULD. 2) LZ consists of amino acids at positions 500–527. Phosphorylation of Thr501 at LZ can phosphorylate signal transducer and activator of transcription 1 (STAT1). This process requires participation through K48-linked polyubiquitination. 3) 578–619 amino acids form a helix–loop–helix (HLH) structure of IKKε. 4) Amino acids at positions 383–647 form a domain that interacts with human DEAD-box protein 3 (DDX3) to induce Ser172 autophosphorylation of IKKε. 5) C-terminus amino acid of IKK at positions 686–705 constitutes a domain that is closely associated with IFNβ promoter activation. K63-linked polyubiquitination occurs by phosphorylation of IKKε Lys30, Lys401, and Met416. Ubiquitinated IKKε recruits downstream members of the NF-κB pathway. Lysine ubiquitination (K63-linked polyubiquitination) at positions 30 and 401 is involved in the development of tumors. SUMOylation of lysine at 231 activates the NF-κB pathway to prevent apoptosis due to DNA damage. Compared with IKKε v1, IKKε v2 lacked 59 amino acids after position 64, but it still retains kinase activity. However, IKKε v3 lost amino acids from positions 1–85, thus losing kinase activity.

The human IKKε gene is located in the 32.1 region of the long arm of chromosome 1, encoding gene KIAA0151, which is a 3.2-kb DNA fragment (Peters et al., 2000). IKKε gene contains 22 exons and possesses three different isoforms (IKKε v1, IKKε v2, and IKKε v3, as shown in Figure 1) (Chang et al., 2021). IKKε v1 is a full-length coding DNA sequence, while IKKε v2 and IKKε v3 lack exon 20 and exon 3, respectively, due to RNA alternative splicing and editing (Chang et al., 2021). The IKKε v2 lacking the helical domain and missing 59 amino acids after amino acid position 644 still maintains its kinase activity (Chang et al., 2021). The kinase domain of IKKε v3 is defective, and the kinase activity is lost due to the loss of amino acids at positions 1–85 (Chang et al., 2021). Mature IKKε is located in the cytoplasm and contains 716 amino acids (IKKε v1). Specifically including the following domains:

1) A total of 9–300 amino acids form the kinase structure domain (kinase, Figure 1), which can phosphorylate the serine (Ser36, Ser32) of the IκB-α, which will remove the inhibitory effect of the IκB-α on the NF-κB pathway (Shimada et al., 1999). The 38th amino acid (lysine, K38) contributed to the phosphorylation of IκB at the amino acid residues (Ser36). If Lys38 is mutated to alanine (K38A), the IKKε will lose the kinase activity which is critical to the DNA damage–inducible translocation of IKKε to the nuclear bodies (Renner et al., 2010). In addition, the Ser172 of the IKKε is located in the mitogen-activated protein kinase kinase (MAPKK) activation loop, which is an important phosphorylation site for MAPKK, while Ser172 phosphorylation of IKKε is the active form of this kinase (Kishore et al., 2002). Interestingly, there is autophosphorylation of Ser172 once IKKε phosphorylates downstream IkB-α (Shimada et al., 1999).
2) Amino acids at positions 350–383 constitute the ubiquitin-like domain (ULD), which is short but essential for the kinase activity of IKKε. IKKε loses its kinase activity when this region is lost, or when both Leu353 and Phe354 are simultaneously mutated to alanine (Ikeda et al., 2007). It should be noted that the mutation of Leu353 or Phe354 to alanine alone does not affect kinase activity. It is suggested that IKKε could have ULD binding to its kinase domain *via* the surface containing the hydrophobic patch at Leu353 and Phe354 in the ULD. This intramolecular folding is a distinctive characteristic of IKKε (Ikeda et al., 2007). Another function of ULD is to bind IRF3, which is phosphorylated by the kinase domain of IKKε. When IRF3 is phosphorylated, ULD loses its ability to bind IRF3 and the phosphorylated IRF3 enters the nucleus and promotes the transcriptional IFNβ gene expression (Ikeda et al., 2007).
3) Amino acids at positions 383–647 form a domain that interacts with DEAD-box protein 3 (DDX3), which promotes autophosphorylation of IKKε at Ser172 (Kishore et al., 2002). Phosphorylated IKKε could phosphorylate the Ser102 residue of DDX3, which is critical for the recruitment of IRF3 to DDX3 (Gu et al., 2013). DDX3 enhances IKKε phosphorylation, thereby promoting IRF3 phosphorylation and ultimately promoting IFN-β promoter activation, initiating the innate immune response to viruses. DDX3 plays the role of a scaffolding protein (Gu et al., 2013). Notably, certain IKKε inhibitors, such as BX795, inhibit Ser172 phosphorylation of IKKε and inhibit its activation when IKKε is overexpressed. However, this inhibitory effect does not prevent LPS, TNF-α, Poly(I:C), and IL-1α–induced endogenous IKKε phosphorylation of Ser172 (Clark et al., 2009).
4) The leucine zipper (LZ) structure of IKKε is composed of amino acids at positions 500–527, which is present in all members of the IKK family. IFN-β-induced Thr501 phosphorylation of IKKɛ can directly phosphorylate STAT1(Ser708) which could promote transcription of cytokines, contributing to innate immunity (Tenoever et al., 2007; Rajsbaum et al., 2014). This process requires K48-linked polyubiquitination of IKKε (Rajsbaum et al., 2014).
5) IKKε 578–619 amino acids constitute a helix–loop–helix (HLH) structure, but the function of this structure remains unclear (Shen and Hahn, 2010).
6) C-terminal (617–716) of IKKε is crucial for inducing the production of type I interferon. Amino acid deficiency at positions 686–705 of the IKKε C-terminal significantly decreases IFN-β promoter activation (Nakatsu et al., 2014).

Until now, the crystal structure of IKKε has been not elucidated. There is no report on dimerization of IKKε. The function of the leucine zipper and HLH structure had been fully revealed. The structure of IKKε also can be modified by ubiquitination. Certain functions of IKKε require polyubiquitination (e.g., K48-linked and K63-linked polyubiquitination). IKKε can be observed as an oncogene in about 30% of breast cancer patients (Zhou et al., 2013). This is closely related to the regulation of the NF-κB pathway after K63 polyubiquitin modification at the Lys30 and Lys401 positions (Zhou et al., 2013). IKKε polyubiquitinated expression is observed in LPS-treated RAW 264.7 macrophages. Further research has shown that cIAP1/cIAP2/TRAF2 E3 ubiquitin ligase complex contributed to K63-linked polyubiquitination by ubiquitination of IKKε at Lys30, Lys401, and Met416 (Zhou et al., 2013). Polyubiquitinated IKKε recruits and activates the downstream signaling pathway of the NF-κB pathway. If Lys30 or Lys401 (not Lys416) residue of IKKɛ was mutated to alanine, ubiquitination and kinase activity of IKKɛ are decreased (Zhou et al., 2013).

In addition, other studies have shown that SUMOylation modification on Lys231 of IKKε was a process dependent on topoisomerase I–binding arginine/serine-rich protein (TOPORS) which is an E3 ubiquitin ligase (Renner et al., 2010). SUMOylation of Lys231 at IKKε can prevent DNA damage–induced apoptosis (Renner et al., 2010). SUMO-ubiquitination helps locate IKKε in the nucleus. After nucleation, IKKε regulates downstream pathways, phosphorylates nucleosomes to aggregate, and inhibits DNA damage–induced apoptosis (Renner et al., 2010). It is worth noting that reporter gene assays comparing the SUMO-modified IKKε-K231R with the wild-type IKKε found that the SUMO-modified IKKε-K231R still induced interferon-β transcription but lost the ability for entry of IKKε into the nucleus (Renner et al., 2010).

### 2.2 IKKs and IKKε

#### 2.2.1 IKK Family Constitution

**FIGURE 2:**
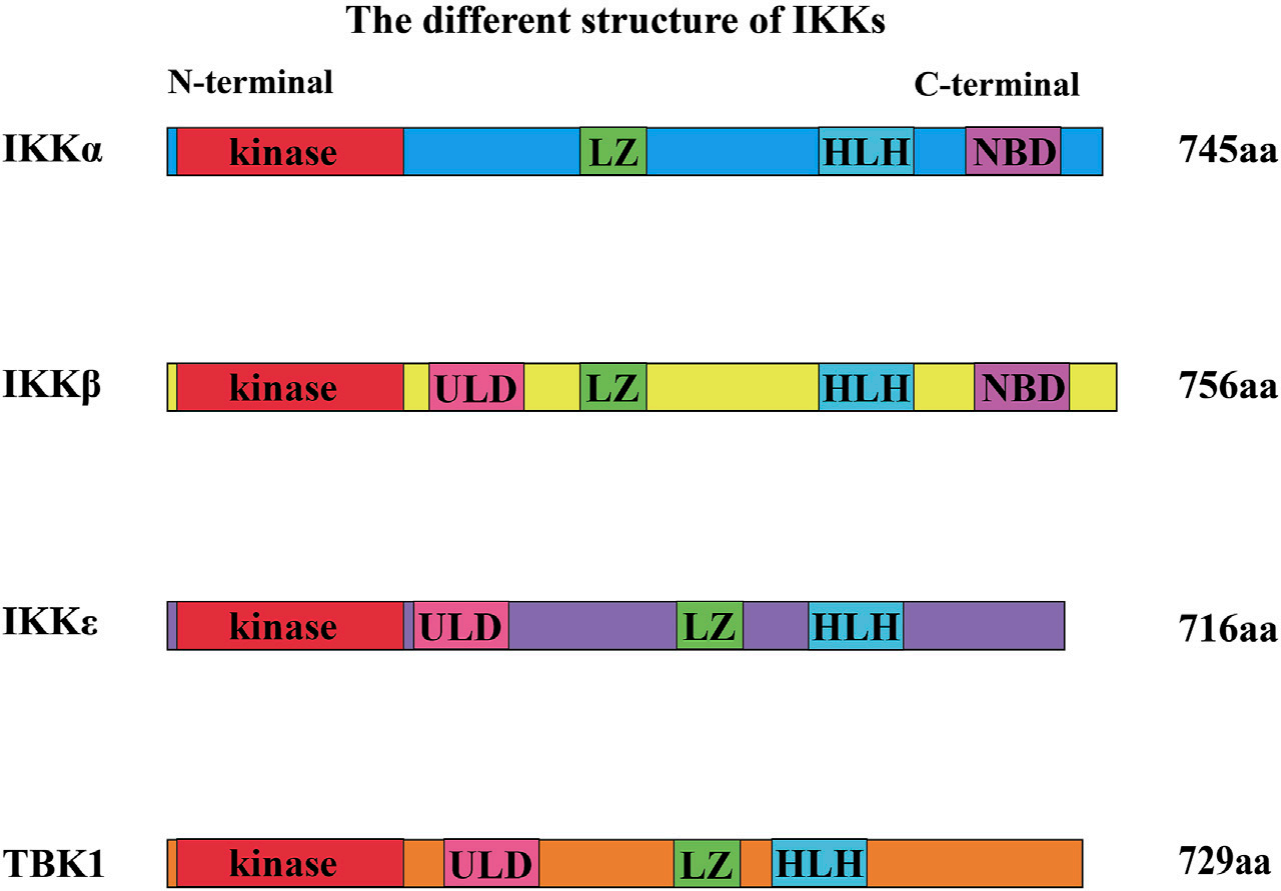
Different structures of IKKs. The N-terminal region of IKKs has a kinase domain, allowing them to trigger the phosphorylation of downstream molecules. They also have a leucine zipper (LZ) and a helix–loop–helix (HLH). Ubiquitin-like domain (ULD) is only discovered in the structure of IKKβ, TBK1, and IKKε. Furthermore, IKKα and IKKβ both have a domain called the NEMO-binding domain (NBD).

IKK family members are classified into canonical family members (namely, IKKα and IKKβ) and noncanonical family members (namely, IKKε and TBK1) (Karin and Delhase, 2000). The members of the IKK family with kinase activity show sequential homology (seen in Figure 2). The N-terminal region of IKKs has a comparable kinase domain, allowing them to trigger the phosphorylation of downstream molecules (Shen and Hahn, 2010). They also have LZ and HLH (Shen and Hahn, 2010). ULD is only discovered in the structure of IKKβ, TBK1, and IKKε. Furthermore, IKKα and IKKβ both have a distinctive domain called the NEMO-binding domain (NBD) (Courtois and Israel, 2011).

IKKα and IKKβ have 51% homologous sequences such as kinase domain, HLH, and LZ (Mercurio et al., 1997; Courtois and Israel, 2011). IKKε and TBK1 also have high sequence homology (Pomerantz and Baltimore, 1999). In addition, the kinase sequence of IKKε shares 27% of the homologous sequence with that of IKKα, but only 24% with that of IKKβ (May et al., 2004; Hiscott et al., 2006; Perkins, 2007; Robinson et al., 2017). Sequence Homology of TBK1 and IKKɛ are 65% in amino acid full-sequences, 65% in ATP-binding region (Reilly et al., 2013), 65% in ubiquitin-like domain (ULD) in mice (Ikeda et al., 2007). In addition, IKKα can also exist in dimer form (noncanonical IKK complexes) (Sun, 2017).

#### 2.2.2 Functional Differentiation of IKKs

IKK members can phosphorylate IκB in the NF-κB pathway, thereby initiating the NF-κB pathway in innate inflammation and tumor. IKKε/TBK1 phosphorylates the Ser36 of the IκB-α subunit, which promotes IκB degradation (Peters et al., 2000). By contrast, IKKβ phosphorylates IκB-α on Ser36 and Ser32, trigging IκB-α degradation (Clément et al., 2008; Zhang et al., 2017). And Ser36 residue is more preferentially phosphorylated than Ser32 by IKKɛ (Shimada et al., 1999).

**FIGURE 3:**
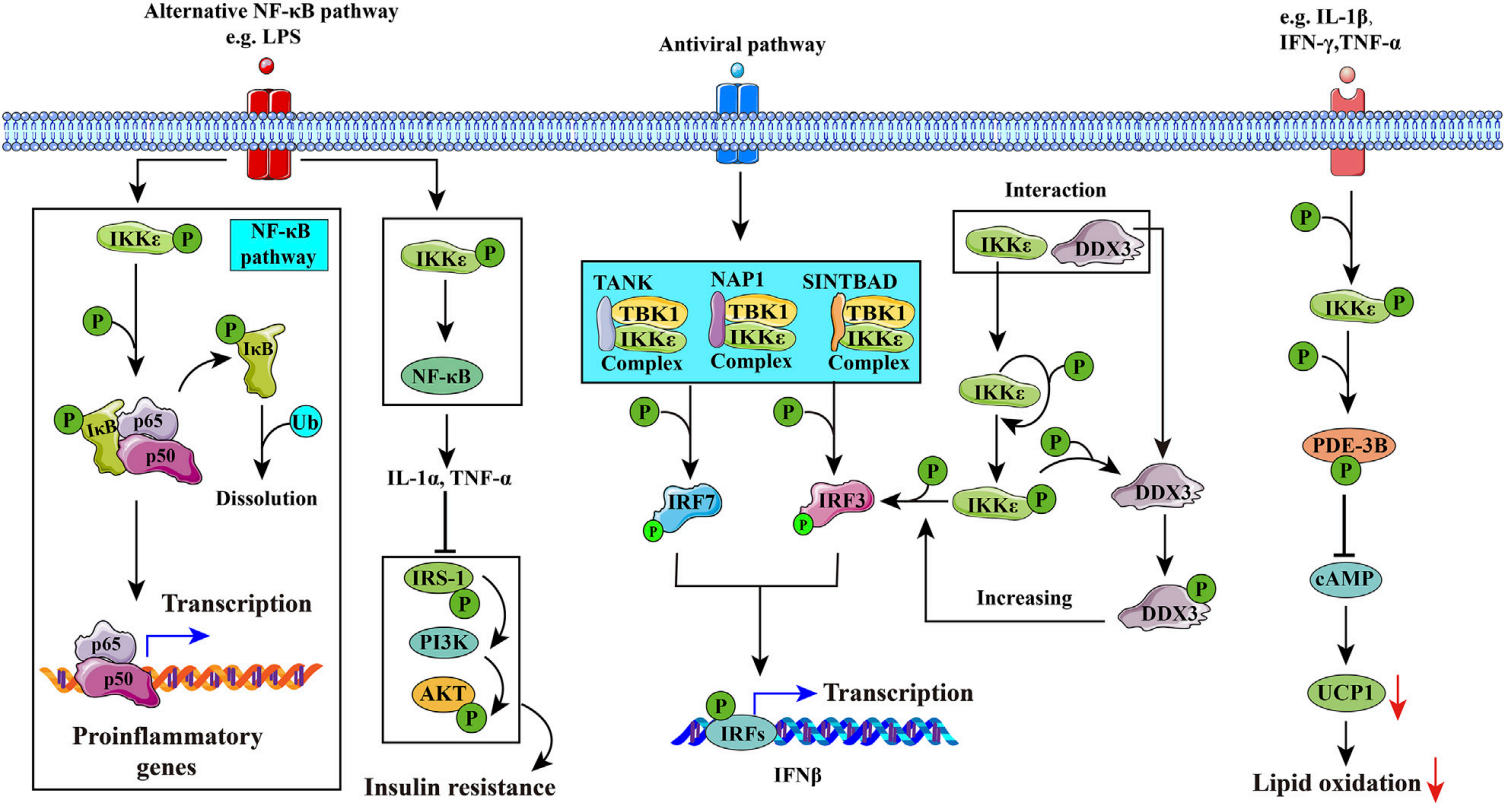
Potential mechanisms of IKKε in diseases. 1) IKKε may induce inflammation through the NF-κB pathway, leading to rheumatoid arthritis. 2) IKKε in hepatic stellate cells (HSCs) activates the NF-κB pathway to secrete cytokines such as IL-1α, which inhibits the insulin signaling pathway in hepatic cells and interferes with glucose and lipid metabolism. 3) IKKε is involved in innate immune responses by phosphorylating interferon regulatory factor 3 (IRF3) and interferon regulatory factor 7 (IRF7). DDX3 enhances the phosphorylation of IKKε and promotes phosphorylation of IRF3, and eventually promotes IFNβ promoter activation. 4) IKKε is activated by PDE-3B phosphorylated at serine 318. Activated PDE-3B reduces cAMP content in adipocytes and inhibits uncoupling protein-1 (UCP1) gene transcription, improving the resistance of adipocytes to catecholamines and inducing obesity.

IKKα and IKKβ both phosphorylate insulin receptor substrate-1 (IRS-1) on Ser312 and inhibit protein kinase B (Akt) (Gao et al., 2002). Targeted disruption of IKKβ in the liver would reverse diet-induced insulin resistance, while systemic insulin resistance results from hepatic overexpression of IKKβ and NF-κB (Yuan et al., 2001; Cai et al., 2005). Overexpression of IKKε or TBK1 also induces a significant increase in Akt phosphorylation at both T308 and S473 and leads to Akt activation (Xie et al., 2011). However, they also play different roles in gene expression, cell growth, and apoptosis (Antonia et al., 2021). IKKε and TBK1 can also form a complex which includes three types: NAP1/IKKε/TBK1 (Pomerantz and Baltimore, 1999; Nomura et al., 2000), TANK/IKKε/TBK1 (Fujita et al., 2003), and SINTBAD/IKKε/TBK1 (Ryzhakov and Randow, 2007), according to different scaffold proteins which they could connect with. IKKε/TBK1 mainly promotes interferon-β transcription (antivirus) and regulates energy metabolism (seen in Figure 3).

#### 2.2.3 Phenotype of IKKs Knockout Mice

There were differential phenotypes in IKKs knockout mice. IKKα^−/−^ mice mostly miscarried during the perinatal period and presented with extensive developmental disorders, such as craniofacial bone malformation and shorter tailbone at E12.5 (12.5 days of gestational age) (Li et al., 1999a). A few IKKα^−/−^ mice survived for 1 month after birth, presenting with thickened epidermis and indistinguishable skin structures (Li et al., 1999a). IKKβ^−/−^ mice showed extensive liver degeneration and apoptosis at E12.5–E14.5 (12.5–14.5 days of gestational age), resulting in embryonic and fetal death (Li et al., 1999b; Tanaka et al., 1999). TBK1^−/−^ mouse embryos showed mild liver degeneration at E13.5, extensive phenotype at E14.5, and prenatal death (Bonnard et al., 2000). IKKε^−/−^ mice were not affected in their life span, but infection-related mortality was significantly higher than was found in wild-type mice, and IKKε^−/−^ mice also showed resistance to HFD-induced obesity (Tenoever et al., 2007; Chiang et al., 2009).

### 2.3 Physiological Functions of IKKε

In a physiological state, IKKε mRNA is expressed the highest in the spleen, human aortic smooth muscle cells (HAOSMC), thymus, peripheral blood white blood cells, placenta, and pancreas (Shimada et al., 1999). In addition, a small amount of IKKε mRNA expression was detected in the lungs, kidneys, prostate, ovaries, colon, and vascular endothelial cells (Shimada et al., 1999; Zhu et al., 2021). There was low expression in the heart, brain, small intestine while little expression was detected in the skeletal muscles and testes (Shimada et al., 1999; Gravel and Servant, 2005). In the liver, IKKε was specifically expressed in stellate cells and not in hepatocytes in the physiological condition but could also be induced to express in hepatocytes by LPS and a high-fat diet (He et al., 2019). In the adipose tissue, under physiological conditions, IKKε was specifically expressed in adipose tissue macrophages (ATMs) and not in the adipocytes (Chiang et al., 2009). Mature IKKε is ubiquitously distributed in the cytoplasm and phosphorylates substrates such as IκBα (Shimada et al., 1999), IRF3 (Sharma et al., 2003), IRF7 (Sharma et al., 2003), PDE-3B (Mowers et al., 2013), DDX3 (Gu et al., 2013), Akt (Zhu et al., 2021), p65 (also known as RelA) (Mattioli et al., 2006), CYLD (Hutti et al., 2009), and YAP (Wang et al., 2017). IKKɛ could also shuttle from cytoplasm into nucleus and phosphorylate nucleosomes while the detailed mechanism is still unclear. (Renner et al., 2010; Rajsbaum et al., 2014). Although IKKε is a member of the IKK family, the NF-κB pathway of the IKKε^−/−^ model is not affected (Hemmi et al., 2004). This suggests that IKKε is not required for the activation of the NF-κB pathway (Shin and Choi, 2019). IKKε deficiency does not affect the classical NF-κB pathway but inhibits LPS-induced C/EBP-δ (CCAAA/enhancer-binding protein-δ) activation and C/EBP-NF-κB–targeted gene transcription (Schwamborn et al., 2003). In addition, IKKε negatively inhibits the NF-κB pathway. In the human IL-17–mediated NF-κB pathway, IKKε phosphorylates nuclear factor kappa-B activator 1 at Ser162 and Ser220 in the signaling complex IL-17R-ACT1-TRAF6, which inhibits the downstream regulation of Act1 and interferes with the downstream NF-κB signaling pathway (Qu et al., 2012). IKKε also phosphorylates p65 (Ser468) which is subsequently shuttled to the nucleus (Mattioli et al., 2006).

### 2.4 Regulation of IKKε Expression

Some common pro-inflammatory factors, such as TNFα, IL-1α, IL-1β, IL-6, IFN-γ, LPS, and peptidoglycan (PGN), can upregulate IKKε gene expression in a variety of cell lines (such as NK cell line and mature B cell line) (Patel et al., 2015). It is noteworthy that the interleukin (IL) family members have different effects on IKKε. IL-4 inhibits the expression of IKKε, while IL-10 does not regulate the expression of IKKε, and IL-17 also promotes the expression and activation of IKKε (Lee et al., 2017), with increased mRNA levels of TBK1, IKKε, IFN-γ, IL-1β, and IL-6 (Lee et al., 2017). TNFα, IL-1β, IFN-γ, and IL-6 stimulated the expression of IKKε in macrophages, but the expression levels of IKKα and IKKβ remained unchanged *in vivo*. TNFα-treated and IL-1β–treated mononuclear macrophages resulted in a three-fold increase in IKKε transcription from baseline (Wang et al., 2005). Increasing the TNFα concentration induced elevation in IKKε expression, which is regulated in a dose-dependent manner (Reilly et al., 2013). T-cell receptor (TCR) is also involved in the regulation of IKKε. When TCR is stimulated, IKKε is activated and further phosphorylates the serine residues (Ser117, Ser151, Ser161, and Ser324) in the N-terminal regulatory domain of nuclear factors of activated T cells (NFATs) to inhibit CD8^+^ T-cell activation, inhibiting T cells by negative feedback (Zhang et al., 2016). In total, IKKε serves as a bridge between pro-inflammatory factors and downstream phosphorylated substrate.

## 3 IKKε in Pathological State

**FIGURE 4:**
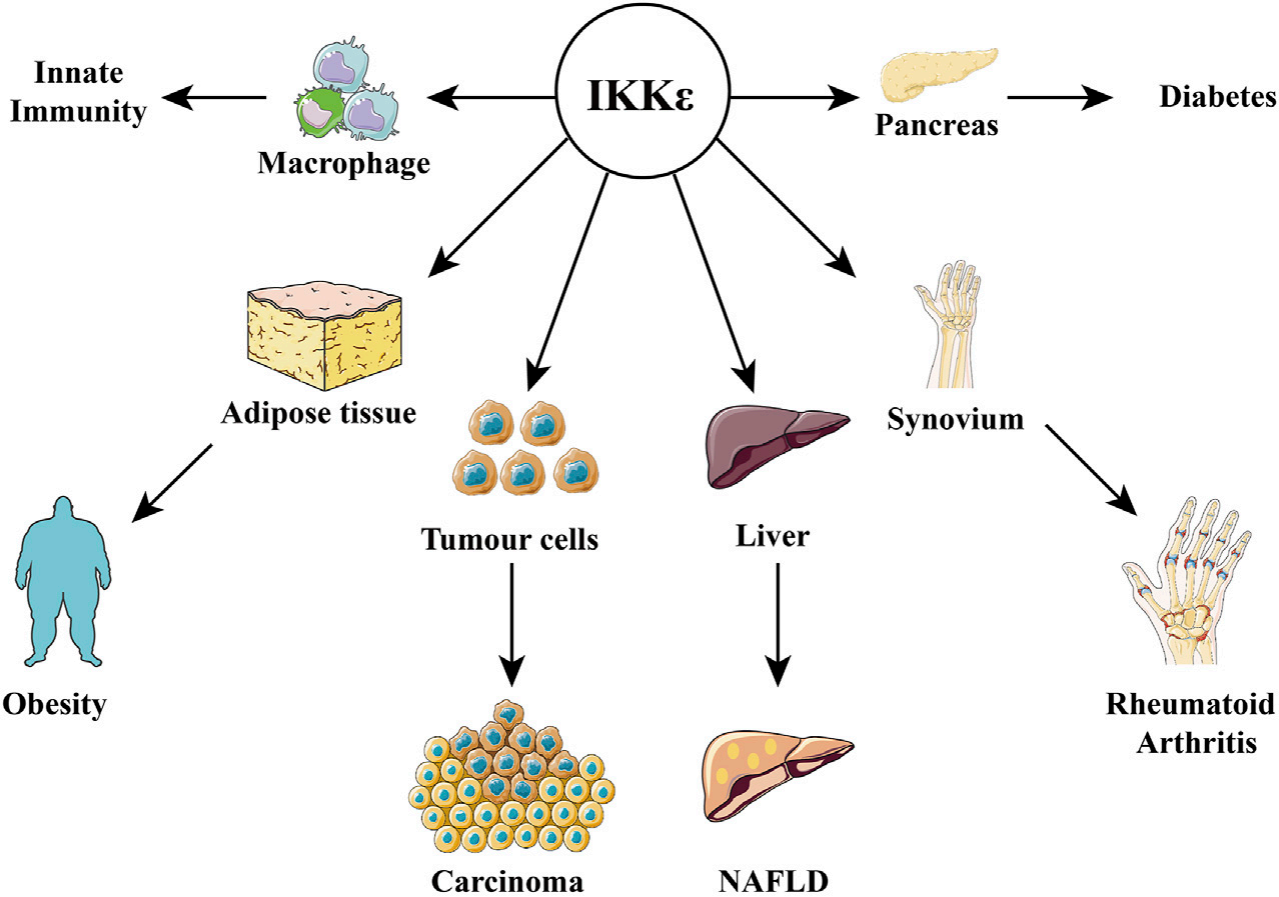
Relationship between IKKε and diseases. IKKε is expressed in a variety of tissues and is therefore involved in the pathophysiological processes of many diseases, including obesity, diabetes, innate immunity, rheumatoid arthritis, cancer, and nonalcoholic fatty liver disease.

In the pathological state, IKKε can be induced to express in more organs under stimulus factors (virus, LPS, and TNF-α). It is highly expressed in synovial cells of rheumatoid arthritis (Sweeney et al., 2005) and malignant tumor cells, such as glioma (Li et al., 2012), esophageal squamous cell carcinoma (Kang et al., 2009), pancreatic ductal adenocarcinoma (Zubair et al., 2016), lung squamous cell carcinoma (Li et al., 2015). It is highly expressed in the adipose tissue of obese patients, islet tissues, and nonalcoholic fatty liver. The following are examples of the important roles of IKKε in metabolic diseases (Figures 3, 4).

### 3.1 Obesity

In the past two decades, a large number of studies have shown that metabolic diseases are related to aberrant activation of the natural immune system, and the activated immune cells contribute to mild inflammation in the adipose tissue, pancreas, and liver, leading to a series of metabolic syndromes (Saltiel and Olefsky, 2017). In the physiological state, the adipocytes do not express IKKε. However, the adipose tissue macrophages (ATMs) commonly reside in the adipose tissues. These ATMs are essential for maintaining the sensitivity of adipocytes to hormones such as insulin. ATMs could induce low inflammation in the adipose tissue of obese animals. When ATMs interact with adipocytes, the mRNA content of IKKε in the adipocytes increase significantly (Sanada et al., 2014). Moreover, this increase was found related to the number or density of the ATMs (Sanada et al., 2014). The number of ATMs in the obese mouse model increased rapidly, and F4/80^+^ CD11c^+^ ATMs were present but not found in the adipose tissue of nonobese mice (Lumeng et al., 2007). The macrophages in adiposity were in two polarization states (M1 and M2), which alternately maintained the sensitivity of the adipocytes to insulin (Saltiel and Olefsky, 2017). In obese mice, M2-polarized macrophages (anti-inflammatory macrophages) were transformed into M1 macrophages (pro-inflammatory macrophages), which released inflammatory factors (such as TNF-α) to induce inflammatory responses (Lumeng et al., 2007). The increased proportion of M1/M2 macrophages is a biomarker of adipose tissue inflammation and is related to insulin resistance and the development of metabolic diseases caused by obesity (Baker et al., 2011).

In addition, adipocytes are involved in inflammatory regulation. A recent study showed that TNF-α–upregulated IKKε expression in adipocytes through microRNA let-7a and protein Lin-28 homolog B (Lin28B) led to resistance to catecholamine-stimulated lipolysis in adipocytes (Li et al., 2019). In obese mice, IKKε/TBK1 could activate and phosphorylate phosphodiesterase 3B (PDE-3B) at Ser318 (Mowers et al., 2013), decreasing the cAMP level and catecholamine-stimulated lipolysis. Also, activated PDE-3B inhibited uncoupling protein-1 (UCP1) gene transcription and reduced fat oxidation (Mowers et al., 2013; Reilly et al., 2015). UCP1 can promote uncoupling of oxidative phosphorylation in the mitochondria and accelerate heat production. Inhibition of UCP1 leads to a decrease in thermogenic response and energy expenditure, which in turn promotes fat deposition in mice and humans (Mowers et al., 2013). Amlexanox (AM) inhibition of IKKε/TBK1 increased cAMP content in adipocytes and promoted IL-6 secretion by adipocytes and preadipocytes in the dorsal subcutaneous and groin of mice through the cAMP/p38-dependent pathway. Then, IL-6 stimulated hepatic STAT3 phosphorylation to inhibit the gene transcription involved in gluconeogenesis and decreased blood glucose (Reilly et al., 2015). However, there is still no evidence that AM could directly affect the hepatic IKKε/TBK1 pathway.

Notably, the regulation of IKKε on energy balance does not occur in mice fed with a chow diet (Chiang et al., 2009). There was no significant difference in bodyweight between systemic IKKε deletion (IKKε^−/−^) mice and wild-type mice when they were fed a chow diet, although IKKε^−/−^ mice showed lower triglycerides and higher fasting insulin levels than the control (Chiang et al., 2009). This may be due to the low level of inflammatory responses in the adipose tissue on the chow diet, and therefore no phenotype differences of adiposity between IKKε^−/−^ mice and wild-type mice. In addition, different strains of IKKε^−/−^ mice (C57BL/6 and 129 background) showed different changes in bodyweight and insulin sensitivity in response to different feeding strategies (Yuan et al., 2001; Scheja et al., 2011). These results perhaps were due to other unknown genes regulated by IKKε.

Interestingly, some other studies suggested that IKKε limited meta-inflammation response to overnutrition. Meta-inflammation was termed metabolically triggered inflammation; this chronic state of inflammation is mediated by macrophages located within the colon, liver, muscles, and adipose tissue. Nod-like receptor thermal protein domain–associated protein 3 (NLRP3) inflammasome transcription expression was more significantly increased in M1-activated macrophages from ApoE^−/−^/IKKε^−/−^ than ApoE^−/−^/IKKε^+/+^ with IL-1/LPS stimulation (Patel et al., 2015). ApoE^−/−^/IKKε^−/−^ mice were protected from diet-induced obesity but developed meta-inflammation in the adipose tissue, liver steatosis, and hypercholesterolemia and readily developed atherosclerotic plaques. Moreover, macrophages in the ApoE^−/−^/IKKε^−/−^ mice can be primed for NLRP3 activity following HFD. The transplantation of ApoE^−/−^/IKKε^+/+^ bone marrow to ApoE^−/−^/IKKε^−/−^ mice prevented double knockout mice from developing HFD-induced obesity, and the inflammasome and inflammatory response in the adipose tissue were reduced (Patel et al., 2015). Furthermore, ApoE^−/−^/IKKε^+/+^ bone marrow transplantation was associated with decreased expression of inflammatory factors (NLRP3 and IL-1β) in the liver (Patel et al., 2015). Another study suggested that IKKε-deficient M1 macrophages showed a stronger inflammatory response (NLRP3 pathway activation) to inflammatory cytokines than wild-type cells (from C57BL/6 mice) (Fischer et al., 2021). In general, IKKε is identified as a pro-inflammatory gene from cell signaling transduction. However, the anti-inflammatory function of IKKε in macrophages has been validated. The possible explanation is that IKKɛ may play a different role in various tissues or cell types, or it simply compensates the inflammatory injury, not initiating the inflammation pathway.

### 3.2 Nonalcoholic Fatty Liver Disease

Nonalcoholic fatty liver disease (NAFLD) is a term for a series of diseases, namely, nonalcoholic steatohepatitis, cirrhosis, liver fibrosis, and liver cancer (Tilg et al., 2017). LPS increased IKKε expression in the liver of the LPS+HFD–induced NAFLD mice model, in an LPS dose-independent manner. Long-term low-dose LPS+HFD–induced mice (18 weeks, 125 μg/kg^−1^ day^−1^) were more likely to develop lipid-deposition–induced steatohepatitis and had a more prominent NAFLD phenotype than HFD mice and high-dose LPS+HFD–induced mice (18 weeks, 250 μg/kg^−1^ day^−1^). In addition, this induction can be mitigated by amlexanox (AM) (He et al., 2020). Interestingly, hepatic IKKε expression existed in hepatic stellate cells (HSCs). AM enhanced the expression of insulin-IRS-1-Akt by inhibiting the inflammatory response of HSCs (IKKε-NF-κB-TNF-α/IL-1α) (He et al., 2019). Furthermore, AM promoted the release of IL-6 from the adipose tissues, which phosphorylated STAT3, thereby inhibiting liver gluconeogenesis and reducing blood glucose (Reilly et al., 2015).

In terms of liver fibrosis, mice were treated with a 0.1% diethoxycarbonyl-1,4-dihydrocollidine (DDC) diet for four consecutive weeks to establish a liver fibrosis model. The results showed that phosphorylated IKKε/TBK1 was increased in HSCs (Zhou et al., 2020b). After treatment with AM, mice with hepatobiliary fibrosis showed significantly improved liver function, lower serum AST, and ALT levels and reduced inflammation of liver Kupffer cells (KCs) (Zhou et al., 2020b). During this process, AM inhibited the phosphorylation of IKKε/TBK1 in hepatic Kupffer cells, which may affect the phosphorylation of downstream STAT3. STAT3 phosphorylation and α-SMA expression were decreased when AM was co-incubated with HSCs and TGF-β–activated LX-2 cell lines (hepatic stellate cell) (Zhou et al., 2020b). STAT3 was specifically found in fibroblasts and HSCs, and not in hepatocytes. Therefore, AM inhibited KCs activation and liver fibrosis through IKKε/TBK1. Previous studies have shown that palmitic acid (PA) promoted the transformation of KCs into M1 macrophages (Luo et al., 2017). The expression of arginase 1 (Arg1) and IL-10 in KCs cells treated with PA was increased by AM. Arg1 and IL-10 are markers of polarization of M2 macrophages, indicating that KCs are transformed into M2 macrophages under AM intervention (Zhou et al., 2020a). In addition, the therapeutic effect of AM on NAFLD also depended on the activation of M1. The content of activator protein-1 (AP-1) in subcutaneous fat of patients with obesity and type 2 diabetes was higher (Oral et al., 2017). AP-1 is a transcription regulatory factor of M1 and is involved in obesity-related adipose tissue inflammation. AP-1 and inflammatory factors transcription were decreased following AM treatment, suggesting that AM reduced the M1/M2 ratio in adipose cells and inhibited the inflammatory response in adipose tissue (Oral et al., 2017).

### 3.3 Diabetes

IKKε is involved in pancreatic β-cell regeneration in animal models of type 1 diabetes (T1D). Xu et al. (2018) found that cinnamic acid derivative (E)-3-(3-phenylbenzo[c]isoxazol-5-yl)acrylic acid (abbreviated as PIAA) inhibited IKKε/TBK1 and stimulated cAMP-dependent protein kinase A (PKA). Mitosis of islet β cells was subsequently promoted through the cAMP/PKA-mTORC1 signaling pathway. The role of IKKε in the pathologic progression and treatment of T1D has also been demonstrated in some clinical trials. One trial found that in newly onset T1D treated by α-1 antitrypsin (AAT), IKKε expression in whole blood cells was inhibited by 50% (Weir et al., 2018). In addition, transcriptome and interactome analysis of pancreatic β cells and peripheral monocytes in T1D also showed that IKKε played an important role in T1D (Safari-Alighiarloo et al., 2020).

From the perspective of a clinical trial, IKKε inhibitors (such as AM) show favorable effects on diabetes. For example, patients with type 2 diabetes (T2D) combined with NAFLD were given AM orally (25 mg three times a day for 2 weeks), titrated to 50 mg three times a day for 10 weeks, and observed for 4 weeks after treatment to ensure patient safety). The expression of UCP1 in subcutaneous fat of patients with type 2 diabetes was increased, and the β 3-adrenergic receptor ADRB3 gene was also highly expressed in the adipose tissue after treatment (Oral et al., 2017). It is worth noting that the hypoglycemic effect of AM seems to be more dependent on the inflammatory response of patients (Oral et al., 2017). A 12-week randomized, double-blind controlled trial was conducted in 42 patients (obese with type 2 diabetes) divided into two groups. In the experimental group, only seven patients (responders) had a decrease of more than 0.5% in A_1c_ (HbA_1c_). When the gene expressions in the adipose tissue of responders and nonresponders were compared, it showed that the responders had higher expressions of inflammatory factors before treatment (such as FOSB, FOSL1, and AP-1) (Oral et al., 2017). These results indicated that AM played a therapeutic role in diabetes by inhibiting IKKε/TBK1. Notably, there was no significant difference in bodyweight between the placebo group and the experimental group (namely, responder and nonresponder) (Oral et al., 2017). Body fat percentage, limb fat percentage, and muscle percentage did not change, but liver fat content was decreased while serum HDL content was increased in the experimental group (Oral et al., 2017).

## 4 IKKε Inhibitors

In 2006, Bamborough et al. found a compound that specifically inhibits IKKε/TBK1 (5-(1H-benzimidazol-1-yl)-3-alkoxy-2-thiophenecarbonitriles). This inhibitor inhibits the ATP binding site of IKKε (Bamborough et al., 2006). In 2014, Li et al. found three kinds of scaffolds (SR8185, 200A, and 200B) based on 2-amino-4-(30-Cyano-40-pyrrolidine) phenyl pyrimidine in the screening of JNK candidate inhibition (Li et al., 2014). These compounds specifically inhibited the Ser172 phosphorylation of IKKε and showed a significant inhibitory effect on the tumor-bearing mouse model. In addition, the number of novel inhibitors developed based on benzimidazole is also increasing (Johannes et al., 2014; Lefranc et al., 2019). In 2019, Lefranc et al. analyzed 3,050,000 compounds and found that BAY-985 specifically inhibited IKKε/TBK1. Bay-985 is a benzimidazole derivative that competitively inhibits the binding of ATP to the Lys38 of IKKε (Lefranc et al., 2019). It is worth noting that although a variety of inhibitors have been developed (see Table 1), few other inhibitors have been used in clinical trials in addition to AM.

**TABLE 1:** Properties and action sites of IKKε inhibitors.

Name	Action site	IC_50_ (nM)	Specificity on inhibiting IKKε	Structural formula	Reference
BX795	Inhibition specifically on Ser172 phosphorylation of IKKε	41 ± 1	IKKε/TBK1	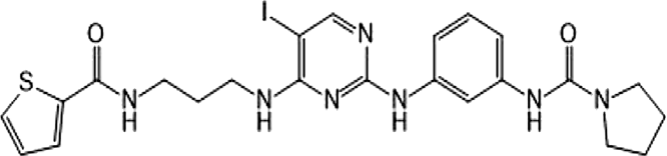	Clark et al. (2009)
Amlexanox	Competitive inhibition of ATP binding sites of IKKε (Lys38)	1,000–2,000	IKKε/TBK1	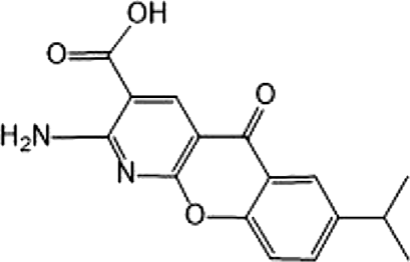	Reilly et al. (2013)
(E)-3 (3-phenylbenzo[c]isoxazol-5-yl)acrylic acid (PIAA)	*	1,070	IKKε/TBK1	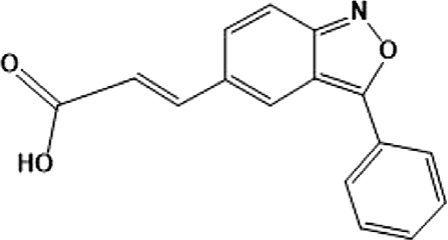	Xu et al. (2018)
SR8185, 200A, 200B	Specifically inhibit phosphorylation of IKKε (Ser172)	*	IKKε/TBK1	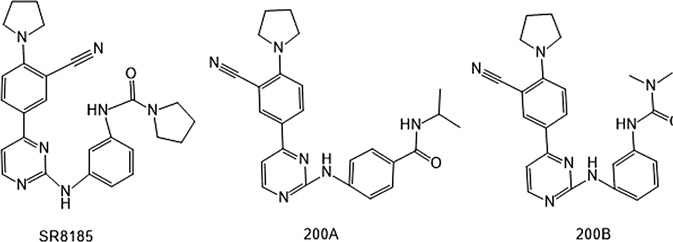	Li et al. (2014)
GSK319347A	*	469	IKKβ/IKKε/TBK1	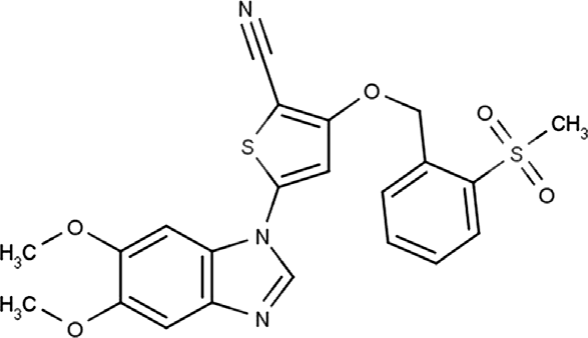	Johannes et al. (2014)
BAY-985	Competitive inhibition of ATP binding sites of IKKε (Lys38)	2	IKKε/TBK1	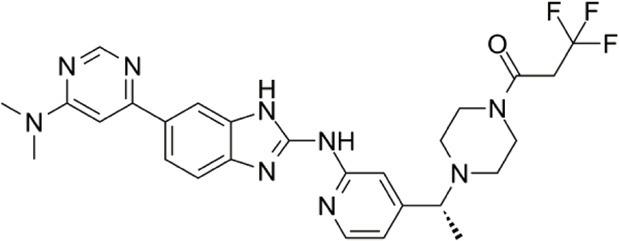	Lefranc et al. (2019)
MRT67307	*	160	IKKε/TBK1	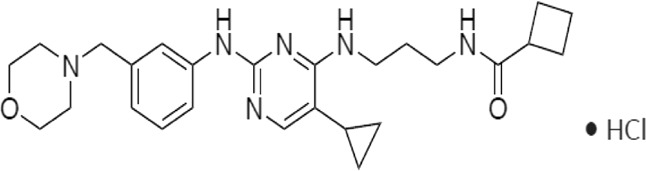	Clark et al. (2011)
CYT387	Decreased IKKε expression at the protein level rather than at the mRNA level	17,680 ± 2,940**	IKKε^#^	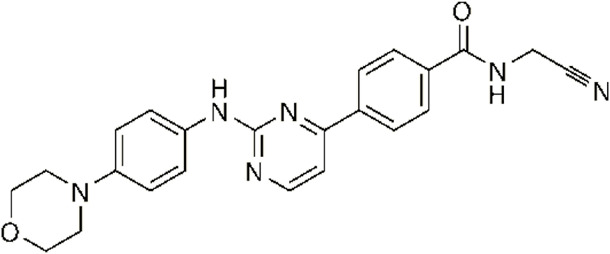	Wang et al. (2021)
TBK1/IKKε-IN-2	*	3.9	IKKε/TBK1	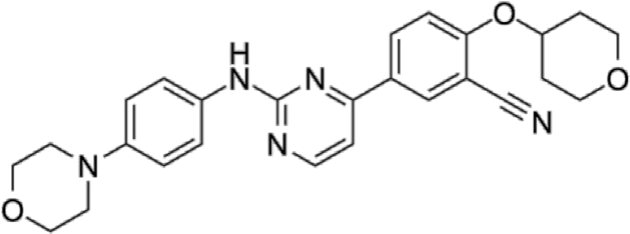	Muvaffak et al. (2014)
TBK1/IKKε-IN-4	*	59	IKKε/TBK1	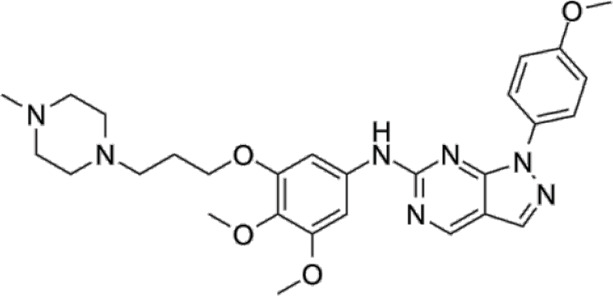	Ou et al. (2011)
TBK1/IKKε-IN-5	*	5.6	IKKε/TBK1	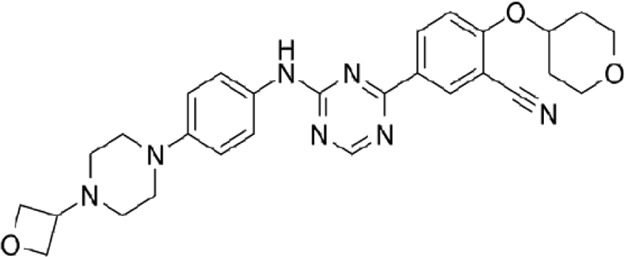	Jenkins et al. (2018)
HPN-01	*	<4.8	IKKα/IKKβ/IKKε	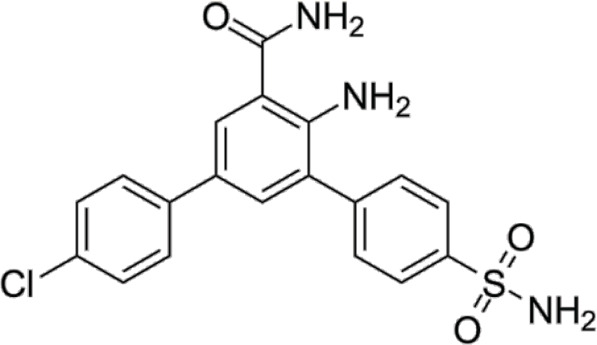	Christopher et al. (2007)
Azabenzimidazole derivatives	*	***	IKKε/TBK1	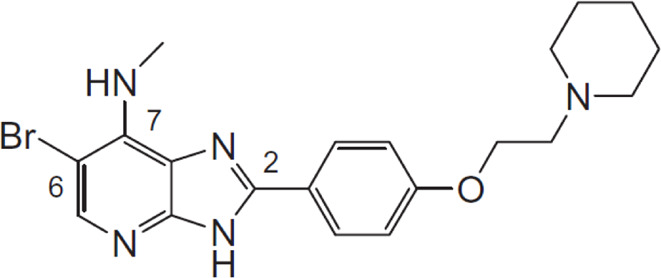	Wang et al. (2012)

*No data; **U87-MG cells for 48 h; *** It has many derivatives possessing different IC_50_; IC_50_, maximum half inhibitory concentration. # No evidence can prove its inhibition effect to TBK1

In 2013, Reilly et al. screened 150,000 compounds and found that AM could specifically inhibit IKKε/TBK1 and lead to IKKε protein suppression (Reilly et al., 2013). AM was first applied clinically in Japan as an anti-asthma drug. In the United States and China, AM was made into an oral patch and used for the treatment of recurrent intractable oral ulcers, but it has been discontinued in clinics in the United States since 2017 (Fu et al., 2012; Uma Maheswari and Shanmugasundaram, 2013; Dosanjh and Won, 2020). Previous studies have shown that AM could be given orally to patients (50 mg three times daily, lasting for 12 weeks) in the treatment of obese type 2 diabetes, showing weight loss, lower blood glucose, and safety (Oral et al., 2017). Notably, the active part of AM lies in its carboxylic acid moiety, and its carboxylic acid derivatives also show strong inhibition of IKKε/TBK1(Beyett et al., 2018a; Beyett et al., 2018b). The carboxyl competition of AM inhibited the activity of IKKε/TBK1 by inhibiting the ATP-binding site of IKKε (Thr156). AM reduced the contents of triglyceride and liver glycogen and inhibited the inflammatory response of the liver and adipose tissue in obese mice. It also decreased serum TNF-α, IL-1α, and MIP-1α and increased the content of the anti-inflammatory factor IL-10 (Reilly et al., 2013). Studies have shown that the liver triglyceride, blood triglyceride, and blood cholesterol contents were decreased significantly in HFD-fed AM-treated mice when compared with the control group (He et al., 2019). AM also increased the expression of adipocyte enrichment proteins (SLC2A4 and PPARγ) and increased insulin sensitivity in mice. AM inhibited the phosphorylation of S6K and S6 in the mammalian target of rapamycin complex 1 (mTORC1) pathway induced by HFD. Correspondingly, insulin-induced phosphorylation of S6 was significantly decreased in IKKε^−/−^3T3-L1 adipocytes. In addition, AM treatment increased the expression of hormone-sensitive lipase (HSL) and UCP1 in brown adipose tissue (Reilly et al., 2013).

The maximum 50% inhibitory concentration (IC_50_) of AM is about 1–2 μM. HFD-induced C57BL/6 mice were administered orally by gavage 25 mg/kg or 100 mg/kg AM (serum concentration was 5 μM) and the results showed that AM prevented HFD-induced obesity within 4 weeks, independent of the dietary intake (Reilly et al., 2013). Interestingly, there was no further weight loss after 4 weeks, even when the AM dose was increased and dietary intake was not changed. The body temperature of mice treated with AM increased by about 1°C when compared with the vehicle (Reilly et al., 2013). However, the weight loss due to AM was reversible, and it lost its inhibitory effect after drug withdrawal (Reilly et al., 2013). Most of the IKKε inhibitors could simultaneously inhibit TBK1. Although the effects of TBK1 and IKKε are similar in structure, there are differences in physiological functions. The loss of IKKε led to increased thermogenesis and insulin sensitivity in animal models (Chiang et al., 2009; Shin and Choi, 2019). Specific ablation of TBK1 in adipocytes reduced HFD-induced obesity but showed glucose intolerance and insulin resistance (Zhao et al., 2018; Shin and Choi, 2019), so specific inhibitors on IKKε should be necessary to avoid the side effects.

Although certain IKKε inhibitors have been discovered, there is no adequate information on IKKε inhibitors on their side effects in clinical practice or mice models. As mentioned in the pathological role of IKKε, IKKε is the key regulator of the IFN-β transcription (Tenoever et al., 2007). Thus, IKKε inhibitors might inhibit the activation of the IFN-β signaling pathway. A previous study showed that although IKKε knockout mice could produce a normal level of IFN-β, they were still hypersusceptible to viral infection because of lacking the IFN-β signaling pathway (Tenoever et al., 2007). Therefore, it could be proposed that patients taking IKKε inhibitors might have undermined immunity and the risk of viral infection. However, AM used as an oral paste of recurrent aphthous ulcers did not show any serious side effects in long-term research and clinical application for decades (Khandwala et al., 1997); it is of great necessity for us to notice its inhibition effect on innate immunity.

## 5 Summary and Prospect

In the past two decades, IKKε has been identified to a certain homology sequence with other IKK family numbers. The physiological function and substrates of IKKε are still not fully revealed. IKKε has been linked with the occurrence and development of obesity, diabetes, and NAFLD. The inhibition of IKKε limited the inflammatory response *in vivo* and improved insulin sensitivity and glucose/lipid metabolism in patients with obesity and diabetes, which proposed a potential therapeutic approach. However, most of the current IKKε inhibitors have low specificity (e.g., combined inhibition of TBK1). Therefore, the development of specific IKKε inhibitors is a challenge and priority for future studies. Furthermore, current studies have shown that IKKε is involved in interferon production and antiviral effects, and systemic overwhelming inhibition on IKKε may lead to susceptibility to viral infection. The development of organ-specific (for adiposity, liver) targeted IKKε inhibitors may help to reduce the side effects of drugs. In conclusion, IKKε plays a pivotal role as a potential therapeutic target in many diseases, especially metabolic diseases, and deserves further investigations in the future.

## Glossary

α-SMA: α-smooth muscle actin
aa: amino acid
Akt: protein kinase B
ALT: alanine aminotransferase
AM: amlexanox
AP-1: activator protein-1
Arg1: arginase 1
AST: aspartate aminotransferase
ATMs: adipose tissue macrophages
CC1: coiled-coil domain 1
CC2: coiled-coil domain 2
C/EBP-δ: CCAAA/enhancer-binding protein-δ
CIA: collagen II–induced rheumatoid arthritis
CYLD: cylindromatosis
DDC: diethoxycarbonyl-1,4-dihydrocollidine
DDX3: DEAD-box protein 3
HFD: high-fat diet
HLH: helix–loop–helix
HSCs: hepatic stellate cells
HSL: hormone-sensitive lipase
IFN-γ: interferon-γ
IFNA 4: interferon alpha-4
IL-1α: interleukin-1α
IL-1β: interleukin-1β
IL-6: interleukin-6
IL-10: interleukin-10
IL-17: interleukin 17
IL-17R: interleukin 17 receptor
IKK: inhibitor of nuclear factor kappa-B kinase
IκBα: inhibitor of nuclear factor kappa-B kinase α
IKKβ: inhibitor of nuclear factor kappa-B kinase β
IKKε: inhibitor of nuclear factor kappa-B kinase ε
IRF3: interferon regulatory Factor 3
IRF7: interferon regulatory Factor 7
IRS-1: insulin receptor substrate-1
KCs: Kupffer cells
Lin28B: protein lin-28 homolog B
LPS: lipopolysaccharide
Lys: lysine
LZ: leucine zipper
MAPKK: mitogen-activated protein kinase kinase
Met: methionine
mTORC1: mammalian target of Rapamycin Complex 1
NAFLD: nonalcoholic fatty liver disease
NAP1: NF-κB essential modifier
NBD: NEMO-binding domain
NFATs: nuclear factors of activated T cells
NF-κB: nuclear factor kappa-B
NLRP3: NOD-like receptor thermal protein domain–associated protein 3
PA: palmitic acid
PDE-3B: phosphodiesterase 3B
PIAA: (E)-3-(3-phenylbenzo[c]isoxazol-5-yl)acrylic acid
PPARγ: peroxisome proliferators-activated receptor γ
SINTBAD: similar to NAP1 TBK1 adapter
STAT1: signal transducer and activator of transcription 1
STAT3: signal transducer and activator of transcription 3
T1D: type 1 diabetes
T2D: type 2 diabetes
TBK1: TANK-binding kinase 1
TCR: T-cell receptor
Thr: threonine
TNF-α: tumor necrosis factor α
TOPORS: topoisomerase I–binding arginine serine-rich protein
TRAF6: TNF receptor–associated factor 6
Ub: ubiquitination
UCP1: uncoupling protein-1
ULD: ubiquitin-like domain
YAP: Yes‐associated protein
Zn: zinc-finger
